# A feed-forward spiking model of shape-coding by IT cells

**DOI:** 10.3389/fpsyg.2014.00481

**Published:** 2014-05-27

**Authors:** August Romeo, Hans Supèr

**Affiliations:** ^1^Department of Basic Psychology, Faculty of Psychology, University of BarcelonaBarcelona, Spain; ^2^Institute for Brain, Cognition and Behavior (IR3C)Barcelona, Spain; ^3^Catalan Institution for Research and Advanced Studies (ICREA)Barcelona, Spain

**Keywords:** spiking model, feed-forward, shape, classifiers, IT

## Abstract

The ability to recognize a shape is linked to figure-ground (FG) organization. Cell preferences appear to be correlated across contrast-polarity reversals and mirror reversals of polygon displays, but not so much across FG reversals. Here we present a network structure which explains both shape-coding by simulated IT cells and suppression of responses to FG reversed stimuli. In our model FG segregation is achieved before shape discrimination, which is itself evidenced by the difference in spiking onsets of a pair of output cells. The studied example also includes feature extraction and illustrates a classification of binary images depending on the dominance of vertical or horizontal borders.

## Introduction

Neurons in the inferior temporal cortex (IT) have been linked to visual shape representation and object recognition (Rolls et al., 1977; Logothetis et al., 1995; DiCarlo and Maunsell, 2000; Riesenhuber and Poggio, 2000; Rollenhagen and Olson, 2000). Lesions in this area result in visual agnosia (Farah, 1990). fMRI studies in humans show how objects activate this part of the cortex and how restricted spots of it are driven by specific classes of stimuli (Desimone, 1991; Malach et al., 1995; Tanaka, 1996). Individual IT cells discriminate, in particular, the shape or color of the stimulus or both parameters (Desimone et al., 1985). Their selective responses are maintained across changes in the size or location on the retina. Actually, in Baylis and Driver's paper (Baylis and Driver, 2001), the visual shape preferences of IT neurons of monkeys were also invariant under two stimulus transformations. The stimuli were different polygon displays and the correlated transforms consisted of either a change in the contrast polarity between the figure and the background or a mirror image. That form of invariance or symmetry is often referred to as “generalization” and its degree of exactness is typically subject to some amount of elasticity.

The exact computational process by which the IT region represents shape remains controversial (Peterson et al., 1991). A central mechanism herein is figure-ground (FG) segmentation, or the segregation of visual information into objects and their surrounding regions (Rubin, 1958). If this task were performed by the brain solely through the contours distinguishing the input displays, then generalization under FG reversal would be expected as well. However, it was absent from Baylis and Driver's results (Baylis and Driver, 2001). Thus, shape coding is not exclusively based on the processing of contour features. For explaining such results, some type of segregation has to be included.

Similarly, psychological findings on human visual shape judgments indicate that one-sided assignment of edges plays a crucial role (Baylis and Driver, 1995a,b; Nakayama et al., 1995; Rubin, 2001). Such an assignment means that the border is “owned” by the side which is imagined “in front,” and regarded as “figure.” Since the dividing curve is the same, the background shares the same informative contour as the original figure, and has its “profile” embedded. Even so, humans typically rate a mirror image of a figure as more similar to the original than the background in isolation (Hoffman and Richards, 1984). Likewise, IT cell responses generalize more strongly across mirror imaging than across FG reversal. That is, they are activated by shape components only after FG assignment (Baylis and Driver, 1995c, see also Hulleman et al., 2005). Apparently, the shape of an object is then coded after the perception of it as a separate entity (however, this issue was contended for a long time and other alternatives were offered, e.g., by Peterson et al., 1991).

We have already favored the idea that the visual system uses one-sided edge assignment to figures (Supèr et al., 2010). In fact, we developed a spiking model which by means of surround inhibition gave FG responses. We concluded that feed-forward connections contribute to the neural mechanisms underlying FG organization, namely, that the phenomenon arises from the computations that happen in earlier stages. Feedback merely controls FG segregation by influencing the neural firing patterns of feed-forward projecting neurons (Supèr and Romeo, 2011). Motivated by all the above observations, we have constructed a network structure, based on our previous work, which explains both the suppression of responses to FG reversed stimuli and the possibility of achieving shape selectivity for the other transformations.

In summary, when an IT cell is selective to a certain shape, the fact that this shape is presented as figure or as ground does matter. We shall be upholding the hypothesis that FG segregation takes place before feature extraction and further processing (alternative hypotheses admitted that shape recognition was possible before FG relationships were determined—Peterson et al., 1991). The present work includes these specific elements: (1) A proposed mechanism for figure segregation: local excitation and global inhibition leading to rebound spiking on regions of smallest area, already introduced by Supèr et al. (2010), and (2) An additional structure for extracting and processing features which, if applied to the considered image type, classifies shapes by vertical|horizontal edge dominance and reproduces the observed weakening in the response when the shape goes into the background.

## Materials and methods

**Figure 1:**
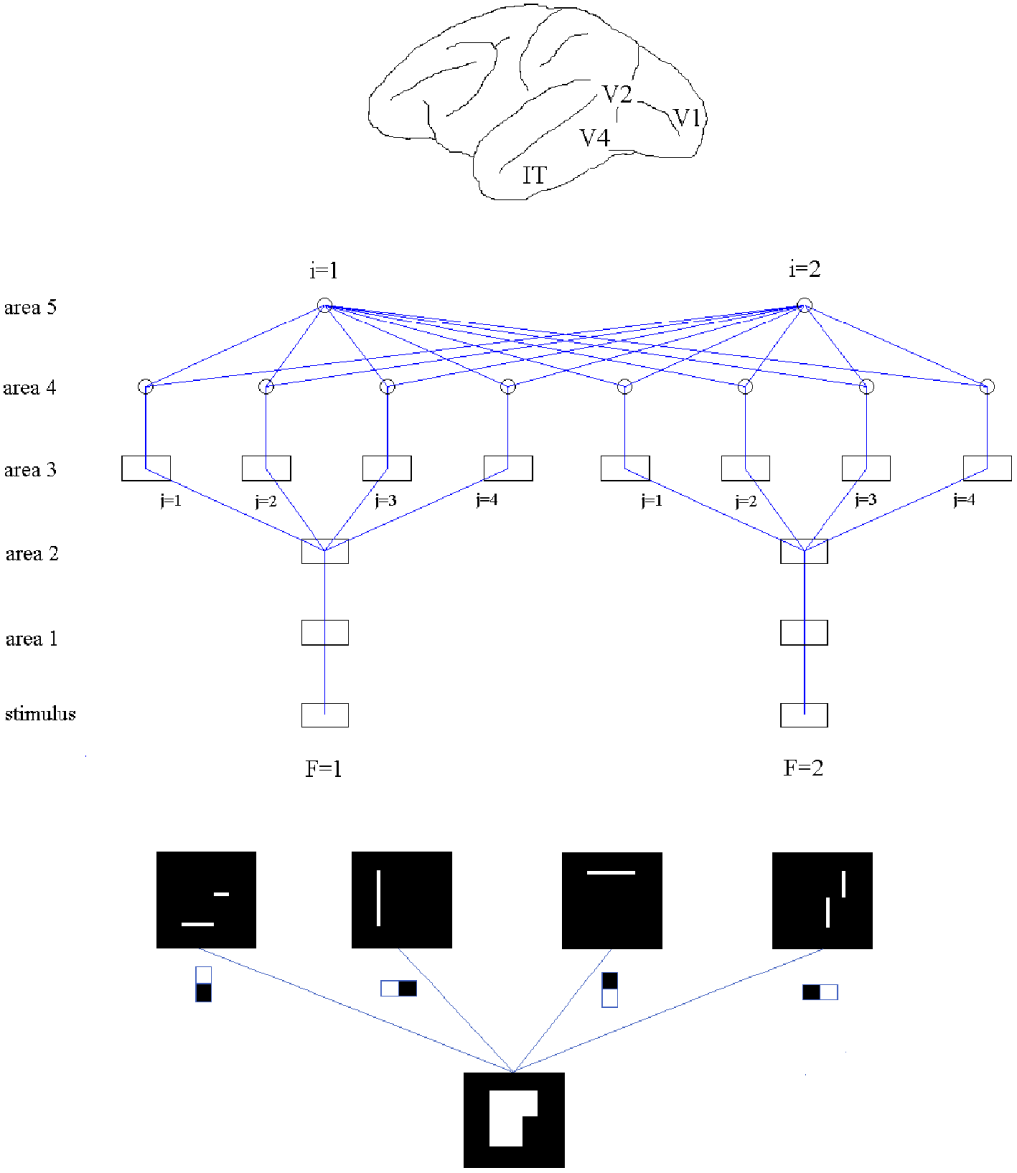
**Top:** Approximate location of V1, V2, V4, and IT in a macaque brain. **Middle:** Structure of the studied network, made of five areas. Areas 1–4 are divided into two “feature” channels which, for areas 3 and 4, are further divided into 4 sub-channels associated with each of the employed receptive fields **f***_j_*, 1 ≤ *j* ≤ 4. Area 5 consists of two neurons. Squares indicate arrays and circles single cells. **Bottom**: An example of feature extraction from a binary array by application of filtering fields (process from area 2 to area 3). The top row show the activated sites when every field is applied.

Our network consists of five areas made of Izhikevich's neurons (Izhikevich, 2003, 2007). The dynamics of that neural model is explained in the Supplementary Material. Of the five areas forming the network, areas 1–4 are divided into two feature channels labeled by *F*, and in areas 3 and 4 each channel is further divided into 4 sub-channels associated with the 4 employed receptive fields labeled by *j*. Area 5 consists of two cells, indicated by *i*, for classification (see Figure 1, middle).

**Figure 2:**
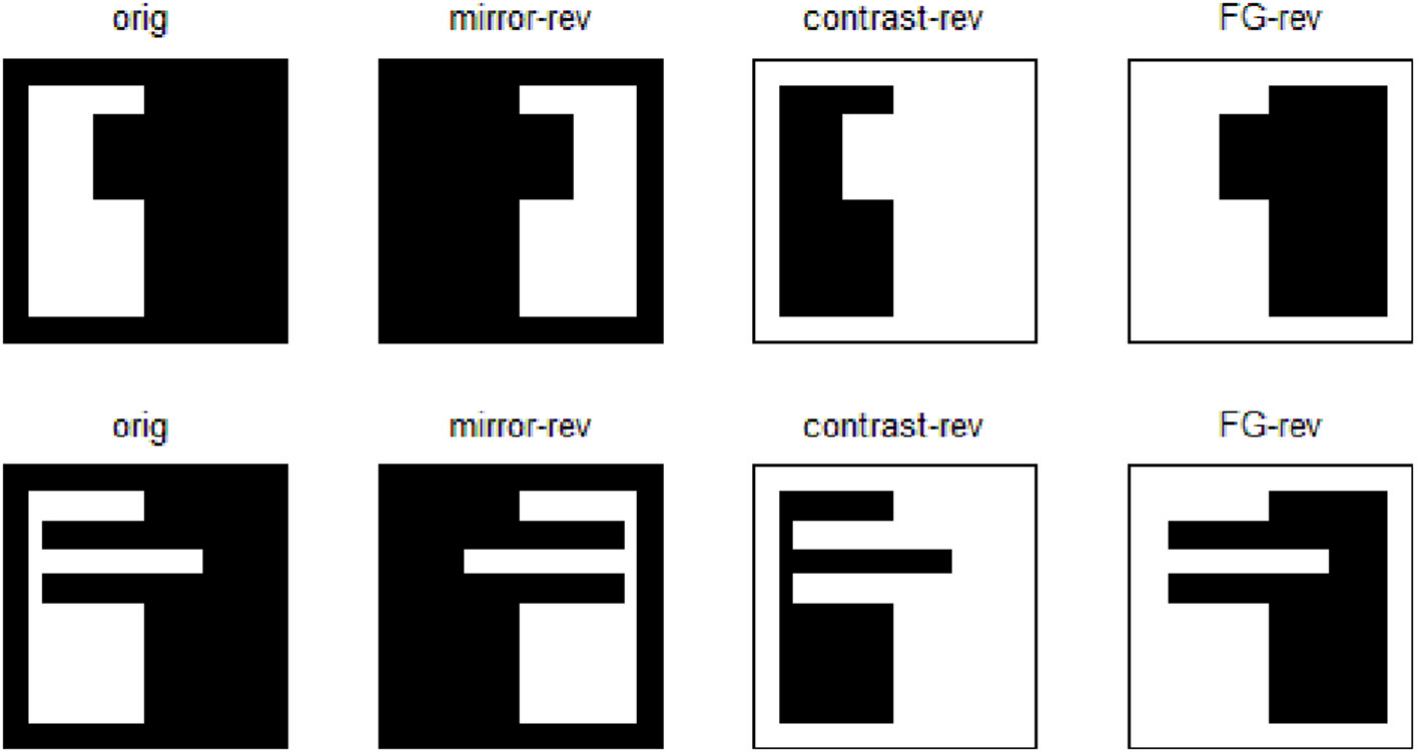
**Chosen images and their mirror-reversals, contrast-reversals, and figure-ground reversals**. Note that within each row, the total length of the existing borders for every image is the same. The two originals have inner size *n* = 64 without margins, outer size *N* = 76 including margins, and an equal area ratio of 0.42 without frame, 0.30 including frame.

The shapes used as stimuli are polygons made of straight frame edges at the top, bottom and along one side, and a “profile” line—possibly but not necessarily curved—on the other side (Baylis and Driver, 2001). When that profile runs between mid-points of opposed frame sides, the total length of the present borders is the same for the original and for the three transformations (see Figure 2).

A combination of local excitation and global inhibition on area 2 is meant to cause the rebound spiking effects described in Supèr et al. (2010). In area 1 the images are accurately represented, as the two-channel input is mapped onto this layer. Only the neurons at the locations of white regions are firing spikes, while those on black regions are quiescent.

Neurons in area 2 receive spiking input from area 1. Each cell gets retinotopic excitatory input and global inhibitory input. For the channel receiving the region of smallest area, the spatial pattern of spiking activity reproduces the excitatory input pattern. On the contrary, for the channel receiving the region of largest area, the spatial activity pattern is the reversal of the input pattern, signaling the complementary region. That change is explained by rebound spiking after a strong inhibition in the smallest region. For neurons on the largest region, global inhibition is partly compensated by retinotopic excitation. However, for cells on the smallest region, that inhibition is the only input and gives rise to a strong a rapid hyperpolarization which provokes rebound spiking of these cells.

The new parts are added “on top” of the previous structure. In area 3, features are extracted by applying a non-linear function—in fact, a step function with given threshold—to convolutions of spike maps and filters (see Figure 1, bottom). The signals produced by application of the different filter types are fed into separate sub-channels. Area 4 collects spatial integrations of the obtained detections within each sub-channel. Finally, area 5, which contains several output units, receives combinations of area 4 signals, including, in principle, all channels and sub-channels. Hypothetically there are as many output units as categories for classification (in our particular example, 2).

The numerical values of our inputs are set by the following rules:

$$\begin{array}{l} I_{1F}\;=w_{1}T_{F},\;F=1,2\\ I_{2F}=w_{2e}S_{1F}-|w_{2i}|\bar{S_{1F}}1,\;\bar{S_{1F}}\equiv \frac{1}{N^{2}}\sum_{k,l}{\left(S_{1F}\right)}_{kl},\;F=1,2\\ I_{3Fj}=w_{3}\Theta (S_{2F}*f_{j}-1),\;F=1,2,\;1\leq j\leq 4\\ I_{4Fj}=w_{4}\bar{S_{3Fj}},\;\bar{S_{3Fj}}\equiv \frac{1}{N^{2}}\sum_{k,l}{\left(S_{3Fj}\right)}_{kl},\;F=1,2,\;1\leq j\leq 4\\ I_{5i}\;=\sum_{F=1}^{2}\sum_{j=1}^{4}w_{5iFj}S_{4Fj},\;i=1,\;2. \end{array}$$

**T***_F_*, *F* = 1,2, stand for original stimulus (*F* = 1) and its contrast-reversed version (*F* = 2). Since the inhibitory weight *w_2i_* is negative, we have written it as *w_2i_* = −|w_2i_|. Concerning the inputs themselves, **I***_1F_*, **I***_2F_*, *F* = 1, 2 and **I***_3Fj_*, *F* = 1, 2, 1 ≤ *j* ≤ 4, are *N × N* matrices; *I_4Fj_*, *F* = 1,2, 1 ≤ *j* ≤4, and *I_5i_*, *i* = 1, 2, are scalars. An analogous convention is employed to indicate the binary (0,1) spike maps: **S***_1F_* denotes the spike map produced by the potentials on area 1 channel *F*, and so on. Thus, **S***_1F_*, **S***_2F_*, *F* = 1, 2, and **S***_3Fj_*, *F* = 1, 2, 1 ≤ *j* ≤ 4, are *N × N* matrices, while *S_4Fj_*, *F* = 1, 2, 1 ≤ *j* ≤ 4, are scalars. For *I* = 1, 2, every *w_5i_* can be regarded as a matrix of two rows, labeled by *F*, and four columns, labeled by *j*. The **1** symbol indicates an *N × N* matrix whose coefficients are all them equal to one. Array convolution product is denoted by the “*” symbol, and Θ indicates the step function Θ (x) = 1 if x = 0 and 0 otherwise. The feature-selective **f***_j_* filters are given by:

$$f_{1}=\left(\begin{array}{c} -1\\ \;1 \end{array}\right)\;f_{2}=(-1\;1)\;f_{3}=\left(\begin{array}{c} \;1\\ -1 \end{array}\right)\;f_{4}=(1\;-1)$$

In the studied set-up we adopt *w*_1_ = 10, *w_2e_* = 400, *w_2i_* = −750, *w*_3_ = 500, *w*_4_ = 5.0, all of them in μ A. The considered images (Figure 2) are squares of side *n* = 64 pixels when margins are not included. As margins are 6 pixels wide, *N* = 76 pixels. The number of white pixels is the same in the two original images, and they yield an area ratio of 0.42 without frame, or 0.30 including frame.

The ability to classify will depend on the particular form of the *w*_5_ matrices. On area 5, cell *i* = 1|2 has to show preference for image 1|2. The question can be addressed by considering the role of the *j* indices, initially labeling the applied filters. For cell 1, limitation to vertical contrast takes place by setting non-zero values in even columns only. Analogously, horizontal contrast for cell 2 is obtained by adopting non-zero values just in the odd columns. Figure 7 illustrates that the strongest signal from FG-reversal goes through *F* = 2, related to the second row of *w_5i_*. Because this signal should yield the weakest output, the remaining non-zero coefficients in the second rows have to be smaller than those in the first rows. A solution meeting this requirement in terms of only two non-zero constants *A*, *B* is

$$w_{51}=\left(\begin{array}{l} \begin{array}{cccc} 0 & A & 0 & A \end{array}\\ \begin{array}{cccc} 0 & B & 0 & B \end{array} \end{array}\right),\;w_{52}=\;\left(\begin{array}{l} \begin{array}{cccc} A & 0 & A & 0 \end{array}\\ \begin{array}{cccc} B & 0 & B & 0 \end{array} \end{array}\right)$$

with *B* smaller than *A*. In practice, satisfactory performance is obtained for *A* = 100 μ A, *B* = 5 μ A.

In agreement with Baylis and Driver's results (Baylis and Driver, 2001) and our previous proposals, FG discrimination is achieved already in area 2, long before shape recognition, and rests on one-sided edge assignment to figures. The shape-selective responses of area 5, identified as IT, depend mainly on the *w_5i_* matrices, which—hypothetically—would consist of a group of learned weights. Shape-coding is evidenced by the difference in spiking onsets for the output units. Cells in V4 code diagnostic boundary features at specific locations, already ascribed to the object figure, which represent through their population response the complete shape. This matches with the findings by Patsupathy and Connor (2002).

## Results

The described model processes sets of figures consisting of original, mirror-reversed, contrast-reversed, and FG-reversed versions of the original one. Depending on the lengths of horizontal and vertical borders, the different activity of the output units classifies the elements of these sets. In addition, responses are similar for original, mirror-reversed and contrast-reversed transformations of the same image, and significantly decrease for the FG-reverse version.

**Figure 3:**
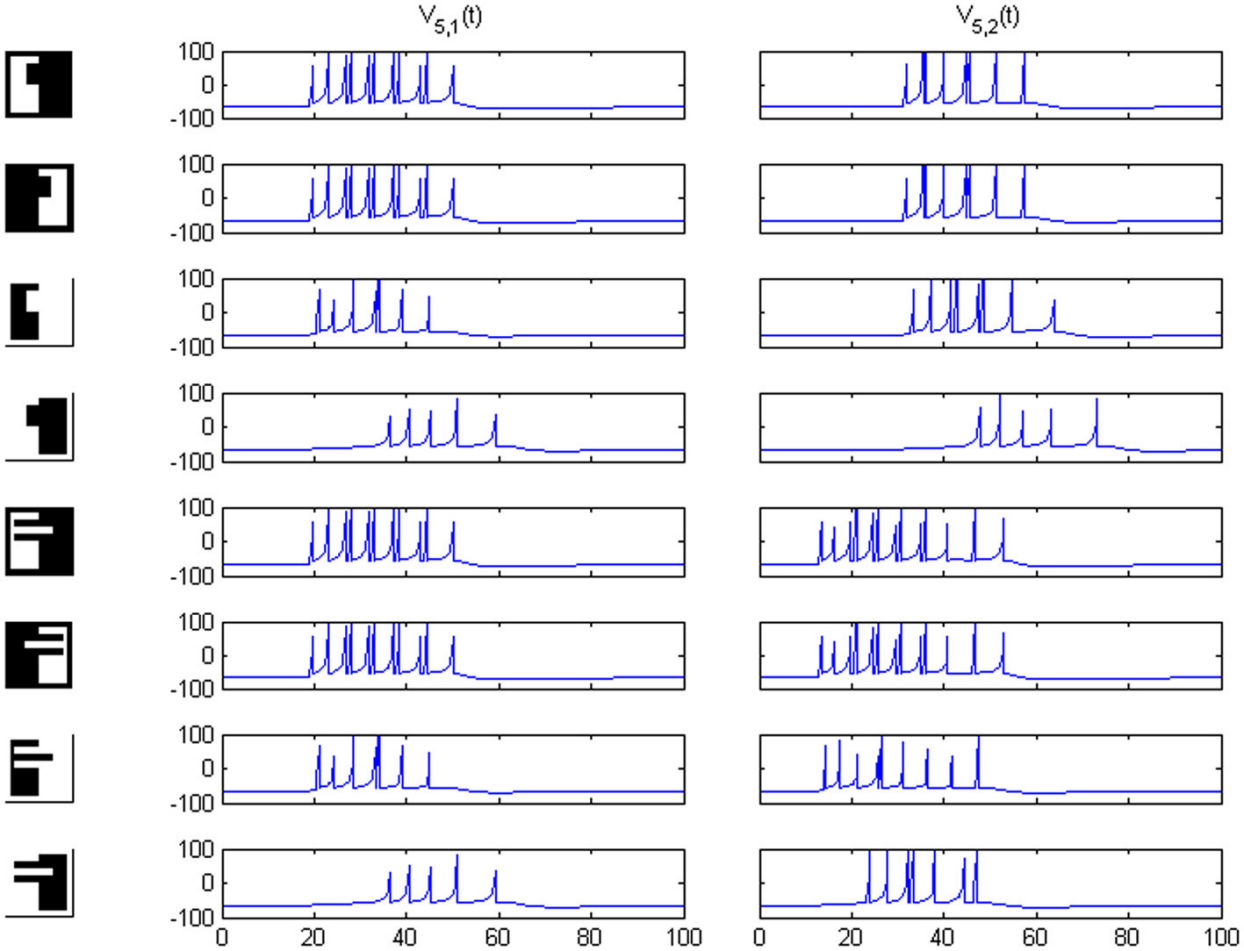
**Network responses on area 5 for the image sets of Figure 2, employing the *w_5i_* weights quoted in the text**. Times are given in ms and potentials in mV. For figure-ground reversal the responses are suppressed while, for the other three cases, the firing order of cells 1 and 2 on area 5 signals the pertinence to one of two possible object categories (second and third columns).

**Figure 4:**
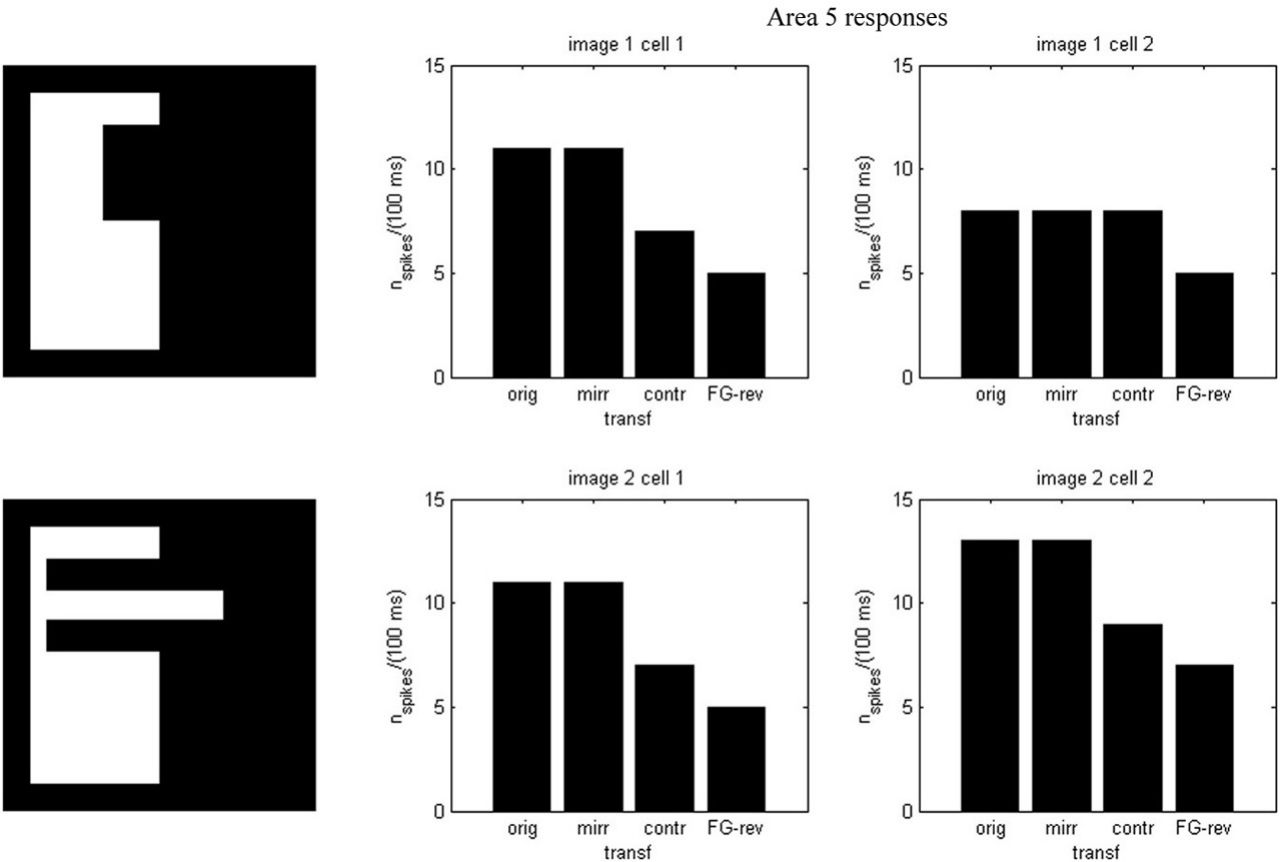
**Spike counts for the example of Figure 2**. Each plot corresponds to an image set and an area 5 cell. In every case there are fewer spikes for FG-reversal.

**Figure 5:**
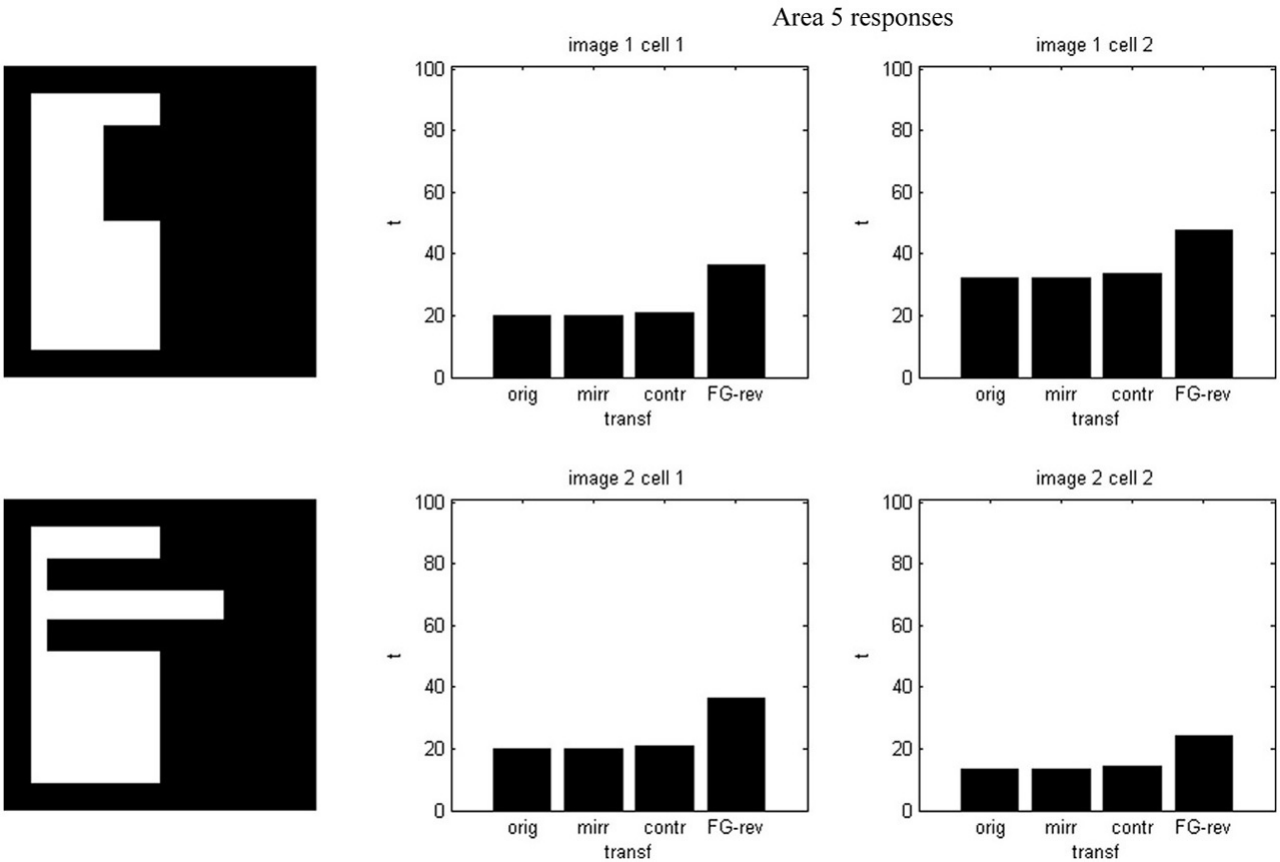
**Firing onset times—i.e., first spike times—for the example of Figure 2**. Each plot is associated with an image set and an area 5 cell. In every set the spiking starts later when FG-reversal is applied.

Results of running the network with our particular matrices are shown in Figure 3. On area 5, cell 1 spikes earlier than cell 2 for image 1 and cell 2 spikes sooner than cell 1 for image 2. Since the non-zero columns of matrices *w*_51_|*w*_52_ correspond to vertical|horizontal contrast features, the employed solution is valid for any case in which the predominance of vertical|horizontal borders can be a distinctive criterion. Moreover, within each image set, responses to FG-reversed images are the lowest because row 2 (which weights the inputs from “*F* = 2” channel) has smaller coefficients than row 1 (which multiplies the “*F* = 1” channel signals). Indeed the spike counts shown in Figure 4 indicate that there are fewer spikes for the FG-reversal of every image. Furthermore, the produced spike bursts start later when applying FG-reversal, as can be seen in Figure 5. On the whole, firing onset times are a better criterion than spike counts.

**Figure 6:**
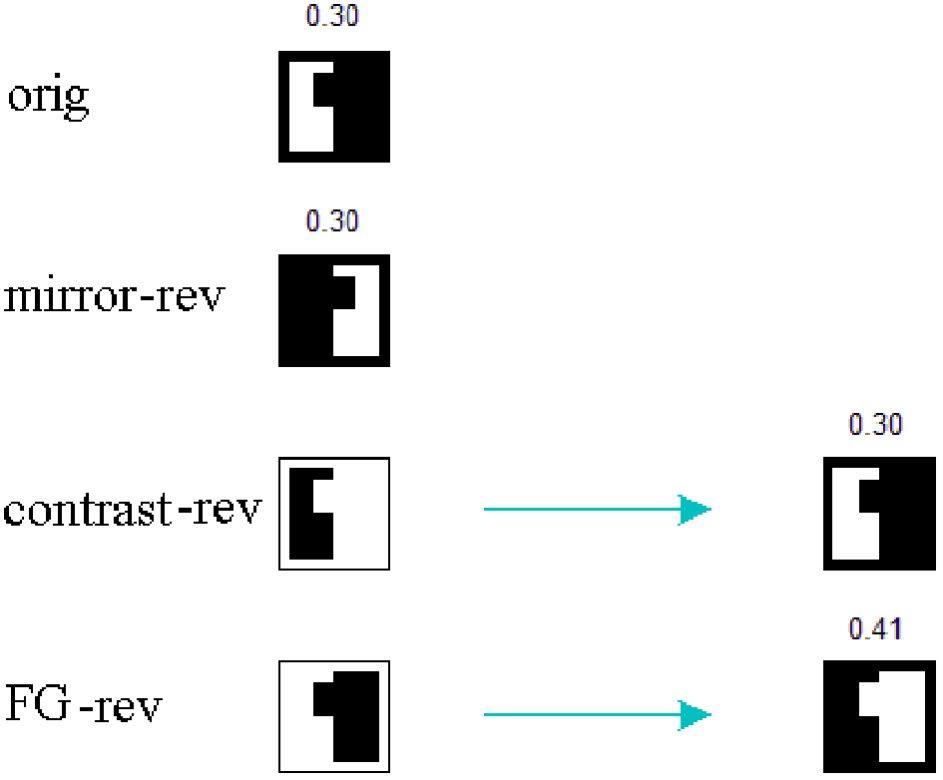
**Spiking area ratios for the figural parts**. The numbers indicate the ratio between spiking area and total area. For contrast and FG-reversal in *F* = 1 channel the figure is segregated after “rebound spiking.” Moreover, in the case of FG-reversal the involved area ratio is the largest one.

**Figure 7:**
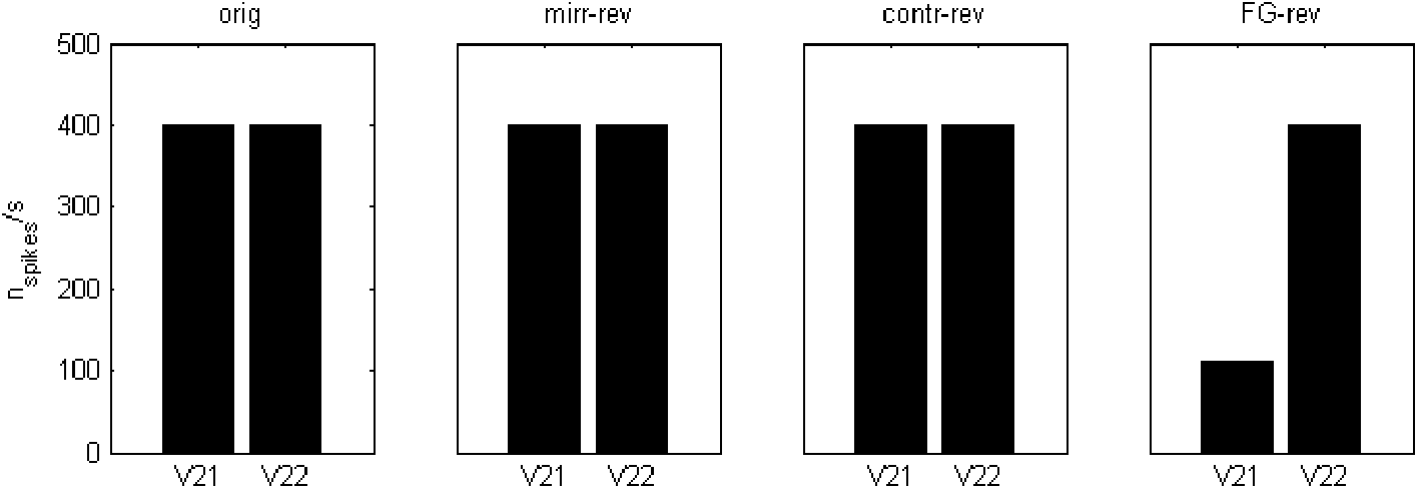
**Spiking rates, in number of spikes per second, for the area 2 potentials V_21_ and V_22_ at a point inside the “figural” region of the first image in Figure 2.** These values were obtained after a 100 ms simulation. In the case of FG-reversal, the spiking for “feature 1” is less frequent than for “feature 2.”

The applied mechanism may be understood in terms of spiking area ratios for figural parts because, in the end, the number of spikes relative to the total area has a decisive contribution to the excitation-inhibition balance. For the case of contrast and FG-reversal in *F* = 1 channel, the figural part is not segregated until “rebound spiking” takes place on area 2 (rebound spiking occurs after a strong inhibition, even in the absence of excitation—see Izhikevich, 2003, 2007 or Supèr et al., 2010). For FG-reversal the involved area is the largest (see Figure 6) and the resulting inhibition, which is proportional to the spiking area, turns out to be somewhat stronger (Figure 7).

**Figure 8:**
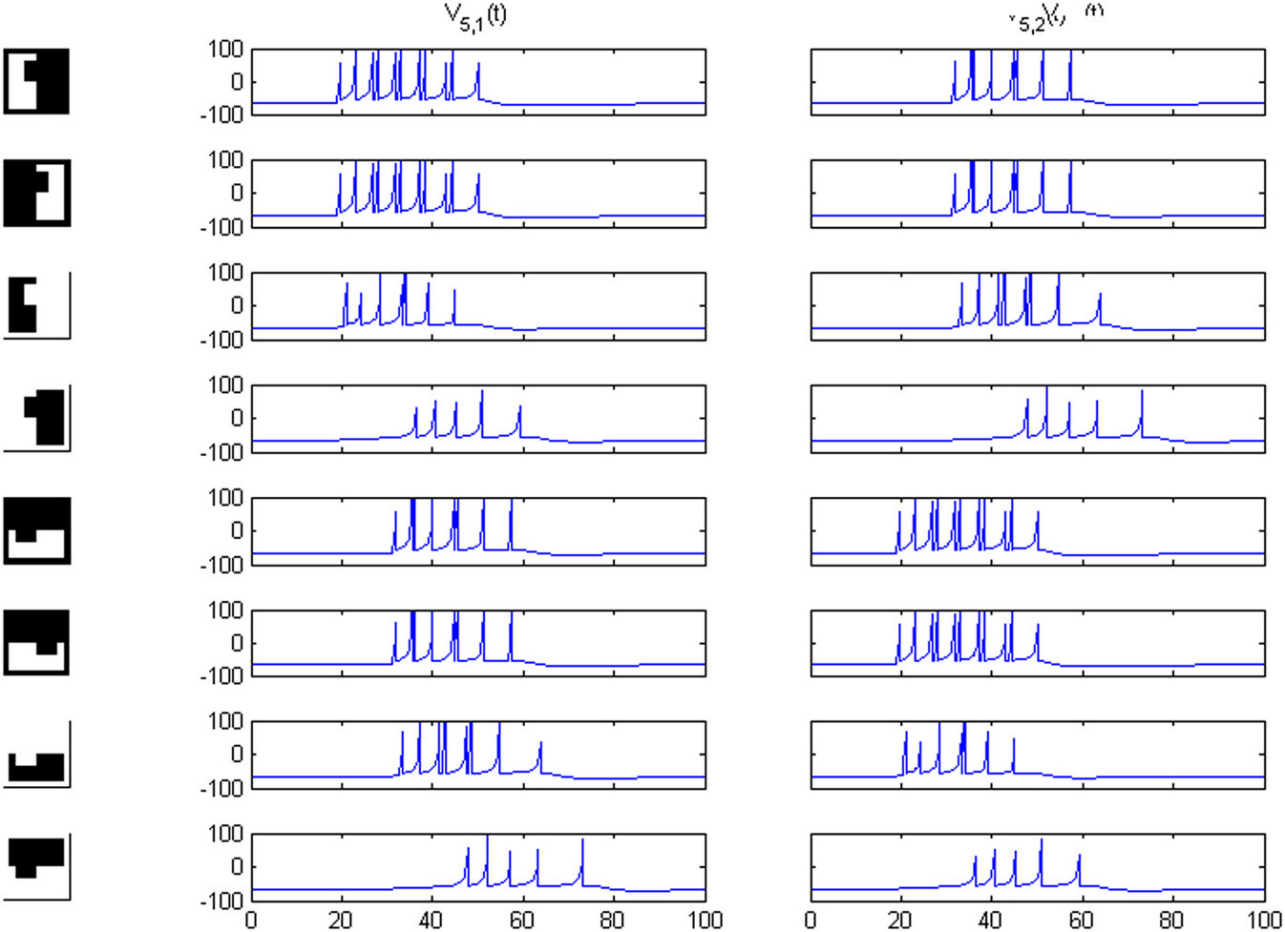
**Potentials on area 5 for the first image set of Figure 2 and its own rotated version**. Cell 1 and cell 2 responses are interchanged.

**Figure 9:**
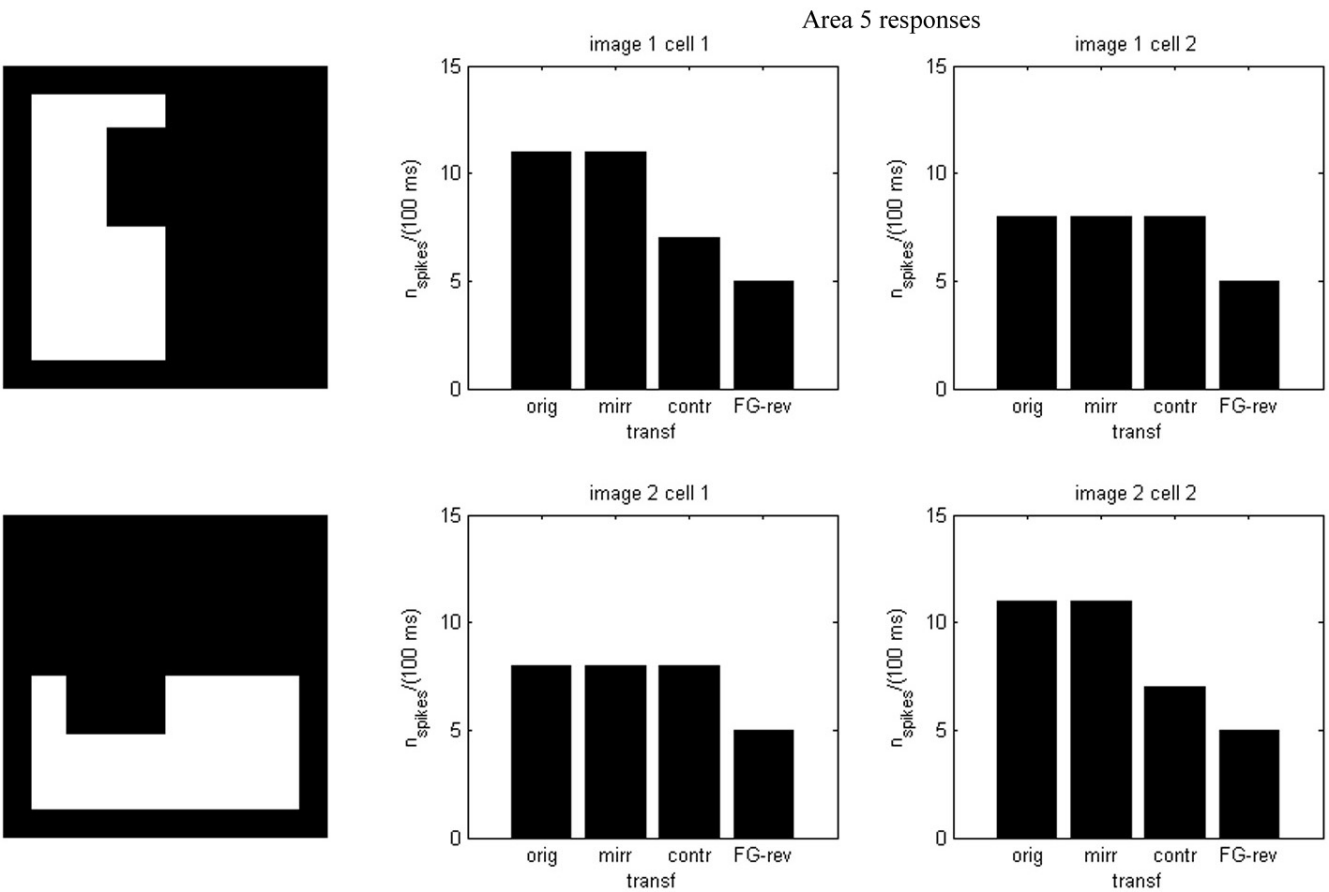
**Spike counts for the images of Figure 8**. Cell 1 and cell 2 counts are interchanged.

**Figure 10:**
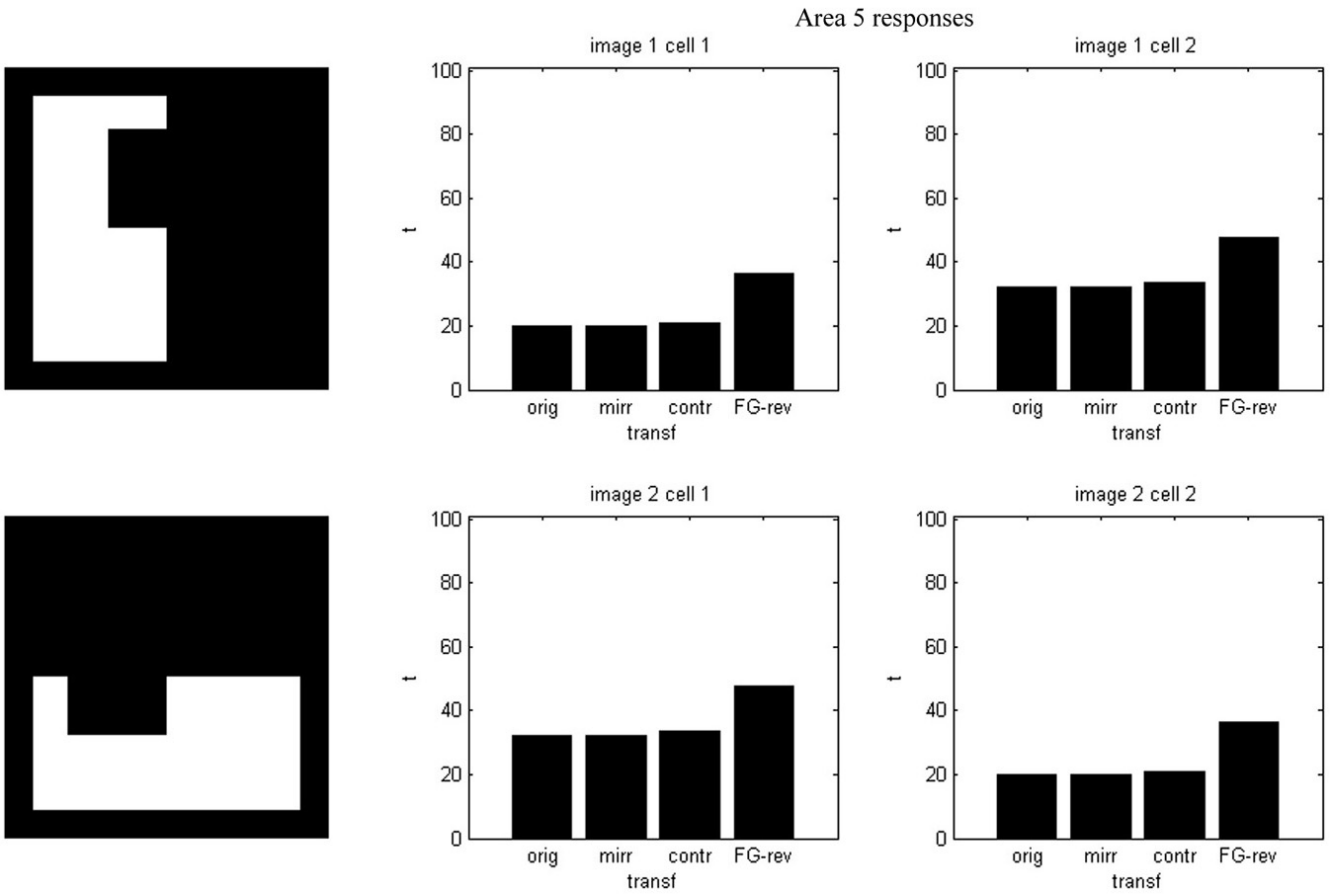
**Firing onset times for the images of Figure 8**. Cell 1 and cell 2 times are interchanged.

Because our criterion rests on differences in length between vertical and horizontal borders, the system distinguishes an image from its own rotated version, as can be seen in Figures 8–10. Predictably, for area 4, responses in sub-channels with even and odd indices are interchanged, and for area 5, the 1 and 2 cell responses are swapped as well.

**Figure 11:**
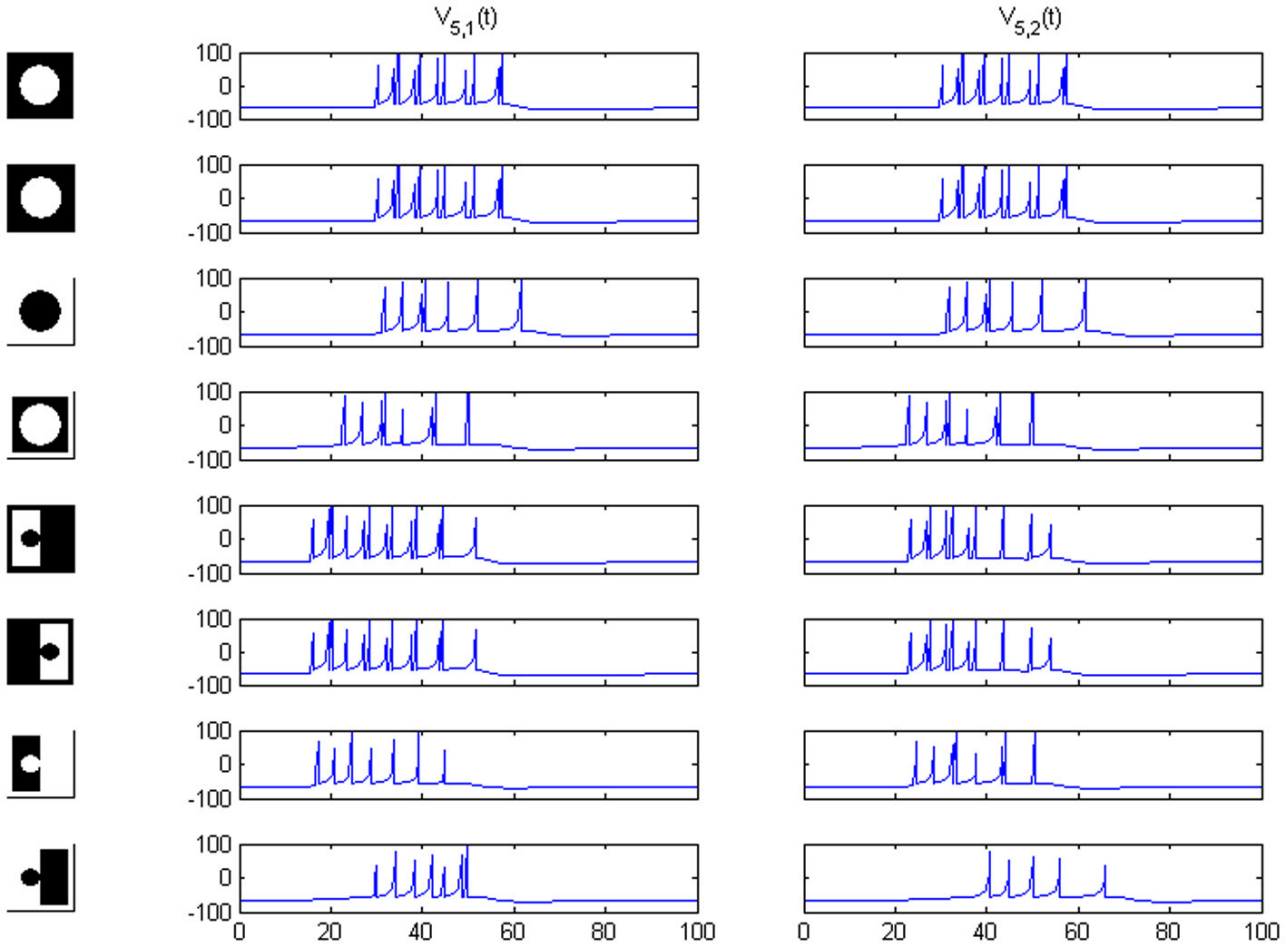
**Rows 1–4: network responses on area 5 for a circle disconnected from a hypothetical frame**. Rows 5–8: responses for a circle connected to the frame by the mid-points of opposed sides (preserving the border length, as required).

In the considered image realm profiles should run between mid-points in opposite frame sides (see lower part of Figure 1 in Baylis and Driver, 2001) in order to preserve the total length of all the boundaries. Going out of this image class we can imagine the case of a disconnected circle. Then, the weakest signal is the “contrast reversed” one, while the “FG-reversed” version produces a higher response (see Figure 11, upper part) caused by the existence of a longer boundary. For this example the third transformation must be simply ignored, because it just amounts to the reversal of an unconnected frame, while the only reasonable analog to FG-reversal is now the contrast reversal itself. Examination of the numerical output reveals that it starts spiking marginally later than the original and mirror-reversal (by 1.25 ms) and with fewer spikes (7 instead of 11). Thus, the result is not inconsistent. When the circular shape is connected to the frame and the overall area ratio correctly set, normal working is restored (Figure 11, lower part).

## Discussion

We have been able to design a network structure which models the suppression of responses to FG reversed stimuli, and shows the possibility of producing selective outputs that generalize across mirror reversed and contrast reversed stimuli. Although the model was not meant for complex images and had no pretence to describe state-of-the-art knowledge on IT processing, it is quite coherent as its outcome fits our previous findings, was constructed using similar values to our forerunning model (Supèr et al., 2010; Supèr and Romeo, 2011) and yields invariance in the pattern of responses across a variety of stimuli and their transformations.

An essential ingredient was the dual pathway for the given figure and its own contrast-reversed version, which represents the existence of two input preferences (Supèr et al., 2010). Although the incoming signals for these two channels are different, the spiking parts in area 2 eventually highlight a single region, identified as “figure.” Despite the space coincidence, the strengths of these signals may still vary, showing a sizable difference for the FG-reversal case. Later, the obtained figural part undergoes a multiple feature extraction process. Spatially-averaged results of that feature detection procedure are then fed into cells mimicking IT neurons. By virtue of the devised scheme, which benefits from the linear character of the *I_5i_* inputs, our IT cells are in fact selective for two image categories. The nature of the performed selection is determined by the weight choice.

A correspondence between model architecture and visual system can be depicted as follows: The first area transforms the input into a spiking train like the Ganglion cell area of the retina, the second area then would be V1, assuming that the LGN (lateral geniculate nucleus) merely relays sensory information. Areas 3–4 may be assimilated to connections occurring both in V2 and in V4, while area 5 would be analogous to IT.

The remarked dependency on orientation can be viewed as the consequence of “experience” (contained in the values of the *w_5i_* weights) that causes the system to perform holistic processing. In the case of the rotated image, the features or components are processed in the same way as in the original (by V4 neurons). If there were edge detectors for enough different orientations and all their outputs could be integrated in a rotationally-invariant fashion, responses for an image and its own rotated version ought to be equal. In our case the limited “experience” implicit in the weights does not suffice for obtaining this symmetry. An implication is that in our model both sorts of information are explicitly encoded as suggested by Schwaninger et al. (2002).

Another consequence would be that our memory of a category has a specific orientation, the usual one in the type of stimulus processed. A well-known example of this affirmation is the Thatcher illusion, where the eyes and mouth of a face are turned upside down (see Thompson, 1980). When the whole image is subsequently inverted the grotesque appearance vanishes. In the context of our model implications, the component representations would then be normal and thus could be matched with the output of the holistic process.

At least for polygons of the studied type, our model bears out the view offered by Baylis and Driver (2001) and provides a computational scheme explaining their observations. FG discrimination is achieved in an area which becomes active before shape selection takes place, and is based on one-sided edge assignments. Such a mechanism, which accounts for the observed generalization, operates by a purely feed-forward process.

### Conflict of interest statement

The authors declare that the research was conducted in the absence of any commercial or financial relationships that could be construed as a potential conflict of interest.
