# Supplementary material for: A feed-forward spiking model of shape-coding by IT cells
Source: Front Psychol. 2014 May 27;5:481. doi: 10.3389/fpsyg.2014.00481 (PMC4034053; doi:10.3389/fpsyg.2014.00481)
Supplement: Supplementary file 1 [file DataSheet1.DOCX]

**Appendix**

Cell dynamics in Izhikevich’s ‘simple model’ (Izhikevich 2003, Izhikevich 2007) is determined by the system of differential equations

supplemented with the after-spike reset rule

The *C, V, u, I* and *t* variables are the membrane capacitance, the membrane potential, the recovery variable, the input intensity and the time, respectively. We assume that typical potential variations are of the order of mV per ms and usual intensities are of a few μA, which means that capacitances should be of the order of μF. For definiteness we adopt *C*= 1μF. The *α, β, γ* symbols indicate fitted constants, with the values *α*= 0.04 μA/(mV)^2^, *β*= 5 μA/(mV), *γ*= 140 μA. The spike limit is set at *V*_sp_ = 30 mV. Regarding the other constants, *a* is a time scale for the evolution of the recovery variable, *b* measures the recovery sensitivity, *c* is the reset value for *V*, and *d* is the height of the reset jump for *u*. We set *a* = 0.02 (ms)^-1^, *b* = 0.25 μA/mV, *c* = −55 mV, *d* = 0.05 μA, which describe the phasic bursting type. As initial conditions at *t_0_* (for us *t_0_* = 0) we use

$$V\text{(}t_{0}\text{)}=-64 \text{mV}, u (t_{0})=b V(t_{0}) \text{ (3)}$$

When viewed as two-dimensional objects, the quantities in eqs. (1)-(3) have to be interpreted as *V→V_kl_*, for *k,l* ranging from 1 to the array size *N*. Spike ‘maps’ are in general binary arrays (including the case of binary scalars when *N*=1) having ones in the sites where the potential is larger than or equal to *V*_sp_, and zeros anywhere else. Numerical integrations were performed by the Euler method, with a time resolution of 0.25 ms. In our simulations *t* ranges from 0 to *t*_max_ = 100 ms.
